# Report of a Case of Polypus Uteri

**Published:** 1849-01

**Authors:** R. C. Hamill

**Affiliations:** Bloomington, Ind.


### ARTICLE VIII.

*Report of a Case of Polypus Uteri.* By Dr. R. C. Hamill, of Bloomington, Ind.

Miss ———, æt. 33, has been suffering under a profuse flux of blood from the uterus for a long time, latterly increasing. I was called first to see her in the fall of 1846. Under treatment, in a short time, she was much better, and I heard nothing more from her until the following May, when I was again sent for. Found her in bed, pale and sallow, conjunctivæ

yellow, tongue coated, pulse small and quick, bowels constipated. Had been suffering for several days from a large sanguinous discharge, accompanied with pain, some coagula. Had been "regular" once in three weeks, the period of menstruation lasting usually about six days, followed by a leucorrheal discharge for the same length of time---menstrua mostly pale. Believing it to be a case of "profuse menstruation," primitive cause unknown, kept up by a want of viotic power, after the use of a few doses of hyd. cum. creta and bi. carb. soda which produced a decided improvement in the secretions, I commenced the use of tonics, elix. vit., and infus. quassia, twice daily, to be continued until within two days of next menstrual period, when the following mist. was administered: tinct. ergot, tinct. hyosc. niger., tinct. ferri. muriatis, equal parts, dose 40 gtt. thrice daily; menstruation came on as was expected; much less pain, less blood; diminished leucorrhea, and the period of the flux shortened. On the ninth day discontinued the mixture. Under the use of tonics between the periods of menstruation, and astringents and opiates during its existence, she improved slightly until the 1st of Sept., when there was a decided change in the symptoms. Menstruation, for the two last periods at the end of the fourth week, came on without much pain, and was not excessive in quantity. At this time, one week before the expected return of the catamenia, a thin, watery, limpid discharge made its appearance in considerable quantities, followed in a few days by a very dark, semi-coagulated, bloody discharge. I requested a vaginal examination, and endeavored to impress upon her mind the absolute necessity of my being in possession of every fact connected with her case, that I might intelligently diagnosticate. She absolutely declined; thought death preferable; requested me to call in another physician in consultation, which was done. The same request made, but declined. She passed the entire winter under the use of palliatives, acidulated drinks, an occasional opiate, &c. Some time during the month of May last, she sent for me again, had had a number of rigors during the last two days, had been bleeding, more or less, for a month every day; œdema of the feet and hands, blood came occasionally "with a dash;" confined mostly to her bed; unwilling still to submit to vaginal examination. Being under the necessity of leaving home for a few weeks, I gave her in charge to Dr. M., a highly intelligent physician of this place. I had frequently told her that I felt confident that a polypus would be discovered upon examination. Dr. M. was of the same opinion. During my absence, the father of Dr. M., a retired physician of deserved reputation, accompanied him. Being very bad, and despairing of aid otherwise, she consented, and he made an examination, and discovered, as expected, a polypus. Nothing was done until my return, a short time after when I determined to remove it. It was situated on the posterior part of the cervix uteri, pear shaped, and about the size of a small orange. Dr. M. assisted me in the operation. I used Gooch's double canula, without the windlass; a small grass line was used for the ligature. Owing to the smallness of the parts, I found much difficulty in applying the ligature. The bleeding, which was pretty free before and during the operation, was measurably checked by the ligature. The cord was tightened twenty-six hours afterward; hemorrhage increased for a short time; patient using cool acidulated drinks. On the next day, complained of pain from tightening the ligature, and slightly feverish; administered a mild purgative, which relieved her. On the fourth day, whilst tying the cord, it broke at the lower end of the canula, I withdrew the upper end of the instrument to the os externum, tied the cord, and replaced it without loosing any advantage. On the sixth day, the separation was complete, and the polypus removed. The patient has been doing well since; menstruating at regular periods, but suffering some from prolapsus uteri.

The body of the polypus was very firm, covered with a kind of loose net work, which appeared to be slightly attached to it and filled with coagulated blood, it might more properly be called a tunica, which, like the tunica vaginalis inclosing the testicle, enclosed the polypus. It is my opinion that this sack became distended with blood and ruptured, thus producing the "gush of blood" which recurred so frequently.

